# Influence of antiresorptive/antiangiogenic therapy on the surgical treatment outcomes of experimentally induced peri-implantitis lesions

**DOI:** 10.1007/s00784-023-05275-w

**Published:** 2023-10-02

**Authors:** Ausra Ramanauskaite, Nadine Krüger, Karina Obreja, Fanya Borchert, Iulia Dahmer, Frank Schwarz

**Affiliations:** Department of Oral Surgery and Implantology, Goethe University, Carolinum, Theodor-Stern-Kai 7, Building 29, 60596 Frankfurt am Main, Germany

**Keywords:** Animal experiment, Peri-implantitis, Antiresorptive therapy, Antiangiogenic therapy, Histological technique, Treatment

## Abstract

**Objective:**

To investigate the influence of various antiresorptive and antiangiogenic medications on the resolution of experimentally induced peri-implantitis lesions after different surgical treatment approaches.

**Materials and methods:**

Forty-eight albino rats randomly received a dual application of the following medications: (1) amino-bisphosphonate (zoledronate (Zo)) (*n* = 8), (2) RANKL inhibitor (denosumab (De)) (*n* = 8), (3) antiangiogenic (bevacizumab (Be)) (*n* = 8), (4) Zo + Be (*n* = 8), (5) De + Be (*n* = 8), or (6) no medication (control (Co)) (*n* = 8). Ligature-induced peri-implantitis lesions were established at 2 maxillary implants over 16 weeks. Afterward, animals were randomly treated either with open flap debridement (OFD) or reconstructive therapy (RT). Treatment procedures were followed by a 12-week healing period. The histological outcomes included residual defect length (DL); defect width (DW) at the bone crest (BC-DW); 25%, 50%, and 75% of the DL; and areas of inflammatory cell infiltrate (ICT). When present, areas of bone sequester (BS) were assessed considering the animal as a statistical unit.

**Results:**

A total of 21 animals were analyzed (Zo: RT = 3, OFD = 1; De: RT = 3, OFD = 2; Be: OFD = 1; Zo + Be: RT = 2, OFD = 2; Co: RT = 3, OFD = 2). Implant loss rates were comparable among the experimental groups. Except for the 25% and 75% DW values that were significantly higher in the Zo + Be group compared to the Co group (*p* = 0.04 and *p* = 0.03, respectively), no significant differences were found among the experimental groups for the DL (lowest—Be: 0.56 mm; highest—Co: 1.05 mm), BC-DW (lowest—De: 0.86 mm, highest—Co: 1.07 mm), 50% DW (lowest—De: 0.86 mm; highest—Be + Zo: 1.29 mm), and ICT (lowest—Be: 0.56 mm^2^; highest—Be + Zo: 1.65 mm^2^). All groups, except for the Zo and Be following RT, showed presence of BS.

**Conclusions:**

The present findings did not reveal a marked effect of various antiresorptive/antiangiogenic medications on the resolution of experimentally induced peri-implantitis lesions, regardless of the surgical approach employed (OFD and RT).

**Clinical relevance:**

Resolution of peri-implantitis lesions may not be affected by the investigated antiresorptive/antiangiogenic medications.

## Introduction

Peri-implantitis is defined as an inflammatory condition affecting dental implants supporting soft and hard tissues and is mainly characterized by progressive marginal bone loss [1, 2]. Substantial evidence supports the disease’s bacterial etiology; therefore, its treatment requires anti-infective therapeutic approaches [3]. Nonsurgical treatment modalities have demonstrated limited predictability in suppressing further disease progression, whereas improved treatment outcomes could be obtained following surgical treatment interventions [4–6].

Managing patients receiving antiresorptive medications, including bisphosphonates (e.g., zoledronic acid) and inhibitors for the receptor activator of nuclear factor-κB ligand (RANKL; e.g., denosumab), or antiangiogenic therapy (e.g., bevacizumab, a vascular endothelial growth factor inhibitor), has become a frequent challenge in clinical practice [7]. As numerous preclinical and clinical studies have shown, the aforementioned drugs considerably suppress bone remodeling, increase bone density, inhibit angiogenesis, and subsequently decrease bone vascularity [8–16]. Given their direct effects on bone metabolism, the occurrence of medication-related osteonecrosis of the jaw (MRONJ) is one of the possible side effects of antiresorptive/antiangiogenic therapy [7]. Although the pathophysiology of MRONJ is still far from being completely understood, emerging clinical data suggest a potential association between MRONJ and the presence of local oral infections (i.e., periodontal or peri-implantitis lesions) that possibly act as local triggering factors for MRONJ’s occurrence and progression [7]. In fact, as pointed out in the findings of a former meta-analysis, patients with MRONJ taking either antiresorptive or antiangiogenic medications were significantly more frequently diagnosed with periodontitis [17]. Furthermore, several case reports have elaborated on MRONJ cases potentially induced by peri-implantitis in patients receiving bisphosphonates [18–20]. On the other hand, one recent experimental study in a rodent model failed to detect any marked effects of the administration of antiresorptive/antiangiogenic medications on the extent of peri-implantitis lesions compared to the controls (i.e., without antiresorptive/antiangiogenic medications), as depicted by similar size and width of the defects as well as the comparable bone microstructure [21].

Currently, there is a lack of data on the potential effect of antiresorptive/antiangiogenic therapy on the effectiveness of surgical therapy of peri-implantitis. Therefore, the main goal of this study was to investigate the influence of antiresorptive/antiangiogenic medications on the resolution of experimentally induced peri-implantitis lesions. It is hypothesized that the treatment outcomes of reconstructive and non-reconstructive peri-implantitis treatments are influenced by various types and common combinations of antiresorptive/antiangiogenic drugs.

## Materials and methods

### Animals

Forty-eight rats of the Wistar strain (age: 6 months, mean weight 0.660 ± 0.56 kg; Janvier Labs, Sulzfeld, Germany) obtained from a certified breeder were used in the study. Animals were housed in appropriately dimensioned cages (2 animals per cage) in a controlled environment with a 12-h light/dark cycle at 22 ± 0.5 °C, 40–70% relative humidity, and were provided water and a special diet *ad libitum*. The study protocol considered the 3Rs (replace, reduce, refine) guidelines for animal experimentation and was approved by the appropriate local authority (Regierungspräsidium Darmstadt, Germany; No.: FU/1232). The following reporting adhered to the ARRIVE Guidelines 2.0 [22].

### Study design and surgical procedures

**Fig. 1:**
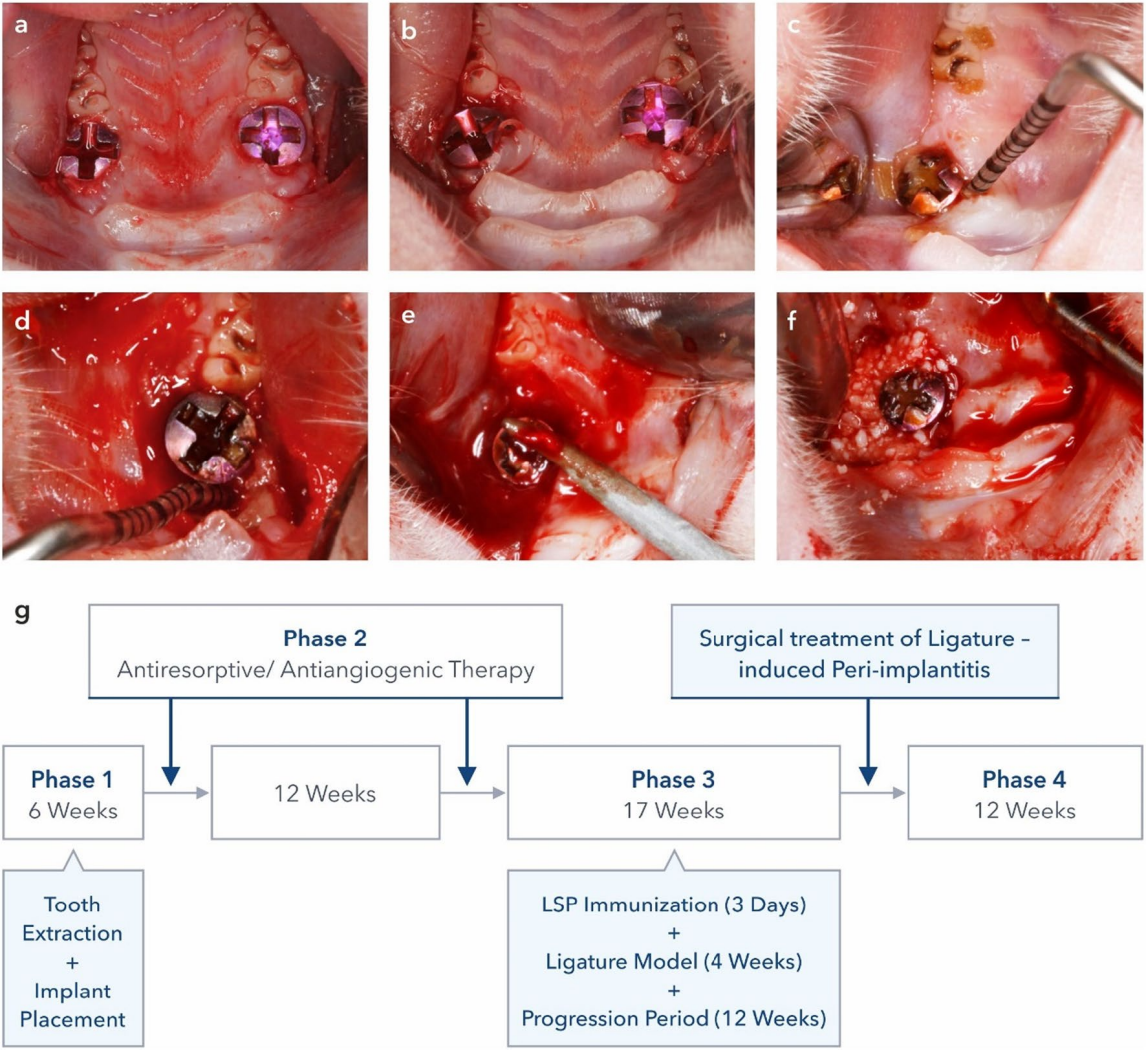
Induction and treatment of peri-implantitis lesions. **a** Situation following immediate implant placement bilaterally in the region of the maxillary first molars. After a 6-week healing period, the first drug administration was provided to the animals in the groups. Drug administration was repeated at 12 weeks. **b** Ligature placement (polyester 6-0) in a submucosal position followed by a single intraperitoneal and repeated topical LPS applications for 3 days. Ligatures were removed after 4 weeks. **c** Clinical signs of inflammation at the end of the progression period at 12 weeks, as indicated by bleeding on probing. **d** Access to peri-implantitis lesion depicting presence of peri-implant intraosseous defect. **e** Treatment of peri-implantitis lesions. Animals were randomly assigned either to the reconstructive OFD or **f** RT treatment. Treatment was followed by the 12-week healing phase. **g** Study outline and timing of experimental phases

The extraction of both maxillary first molars was followed by the immediate insertion of smooth-surfaced titanium mini-implants (Ustomed® Micro-screws, cross, ⌀ 1.2 mm, shortened to 3 mm) [23] at the respective sites. Primary implant stability was obtained in all cases. After 6 weeks of healing, all animals had randomly (block randomization, Randlist, DatInf GmbH, Tübingen, Germany) received the following commonly applied antiresorptive/antiangiogenic medications, including *n* = 8 animals in each group: (1) amino-bisphosphonate (zoledronate 5 mg/kg intravenous, Ribometa® 4 mg/5 ml, Hikma Pharma, Gräfelfing, Germany) (Zo), (2) RANKL inhibitor (denosumab 60 mg/kg subcutaneous, Prolia®, Amgen, Munich, Germany) (De), (3) antiangiogenic medication (bevacizumab 5 mg/kg intravenous, Avastin® 400 mg/16 ml, Roche Pharma, Grenzach-Wyhlen, Germany) (Be), (4) Zo + Be, (5) De + Be, or (6) no medication serving as the control group (Co). Drug administration was repeated at 12 weeks. Subsequently, peri-implantitis lesions were induced by applying an established and validated procedure [21, 24]. The procedure included an intraperitoneal booster lipopolysaccharide (lipopolysaccharide *Escherichia coli* O111:B4, EMD Millipore, Merck, Darmstadt, Germany) injections and daily deposition into the peri-implant sulcus on the buccal and palatal aspects of each implant for 3 days. Afterward, miniature polyester ligatures (Dagrofil 6-0, B. Braun, Melsungen, Germany) were placed in a submarginal position around both implants in each animal for 4 weeks. For this, the ligatures were gently tied in a position directly apical to the mucosal margin while avoiding trauma to the peri-implant tissues, which facilitated the accumulation of a submucosal microbiota and local inflammation that led to a “pocket” formation. This was followed by a progression period of 12 weeks (Fig. 1). During the progression period, a total of *n* = 54 implants (Zo = 8, De = 6, Be = 14, Zo + Be = 8, De+ Be= 12, Co = 6 implants) in 27 animals were lost. Accordingly, a total of *n* = 42 implants (Zo = 8, De = 10, Be = 2, Zo + Be = 8, De + Be = 4, Co = 10) in *n* = 21 animals (Zo = 4, De = 5, Be = 1, Zo + Be = 4, De + Be = 2, Co = 5) further received surgical peri-implantitis treatment.

### Surgical peri-implantitis treatment

After 12 weeks of the disease progression, animals were randomly assigned to either open flap debridement (OFD) or reconstructive treatment (RT) of peri-implantitis. In brief, mucoperiosteal flaps were raised buccally and orally by means of intracrevicular incisions under general anesthesia [25]. Granulation tissues were removed, and implant surface decontamination/debridement was performed using titanium curettes and cotton pellets soaked in sterile saline. In the RT group, peri-implantitis-related defects were filled with a collagenated-bovine-derived xenograft (Bio-Oss Collagen, Geistlich Biomaterials, Wolhusen, Switzerland; Fig. 1). Mucoperiosteal flaps were repositioned to ensure a transmucosal healing phase of 12 weeks. All surgical procedures were performed by experienced surgeons (F.S., K.O., A.R.). During the 12-week healing period, a total of *n* = 28 implants were lost (Co—RT: 2; OFD: 4; Zo—OFD: 2; Zo + Be—RT: 4; OFD: 4; De—RT: 5, ODF: 2; De + Be—RT: 1; OFD: 2).

### Anesthesia protocol

For each surgical intervention, the animals were anesthetized by intraperitoneal injection of 7.5 mg/kg ketamine (Ketanest®, Pfizer Pharma GmbH, Karlsruhe, Germany) and 5 mg/kg xylazine (Rompun®, Bayer HealthCare, Leverkusen, Germany). For postoperative analgesia, 4.5 mg/kg carprofene was administered subcutaneously immediately after surgery, as well as 1, 2, and 3 days postoperatively.

### Histological processing

The animals were euthanized with an overdose of pentobarbitone (Euthanimal 20%, Abbey Laboratories Pty Ltd., Glendenning, Australia) at 100 mg/kg. Jaws were separated and fixed in a 10% neutral buffered formalin solution for 10 days. Tissue blocks were decalcified using an ultrasound-supported water bath and 10% ethylenediaminetetraacetic acid for 12 weeks. Prior to processing and embedding in paraffin, the implants were carefully removed by counterclockwise unscrewing. The two most central serial sections of each block were cut in the horizontal plane with the micrometer set at 3–4 μm and were manually stained with hematoxylin and eosin.

### Histological and histomorphometrical analysis

**Fig. 2:**
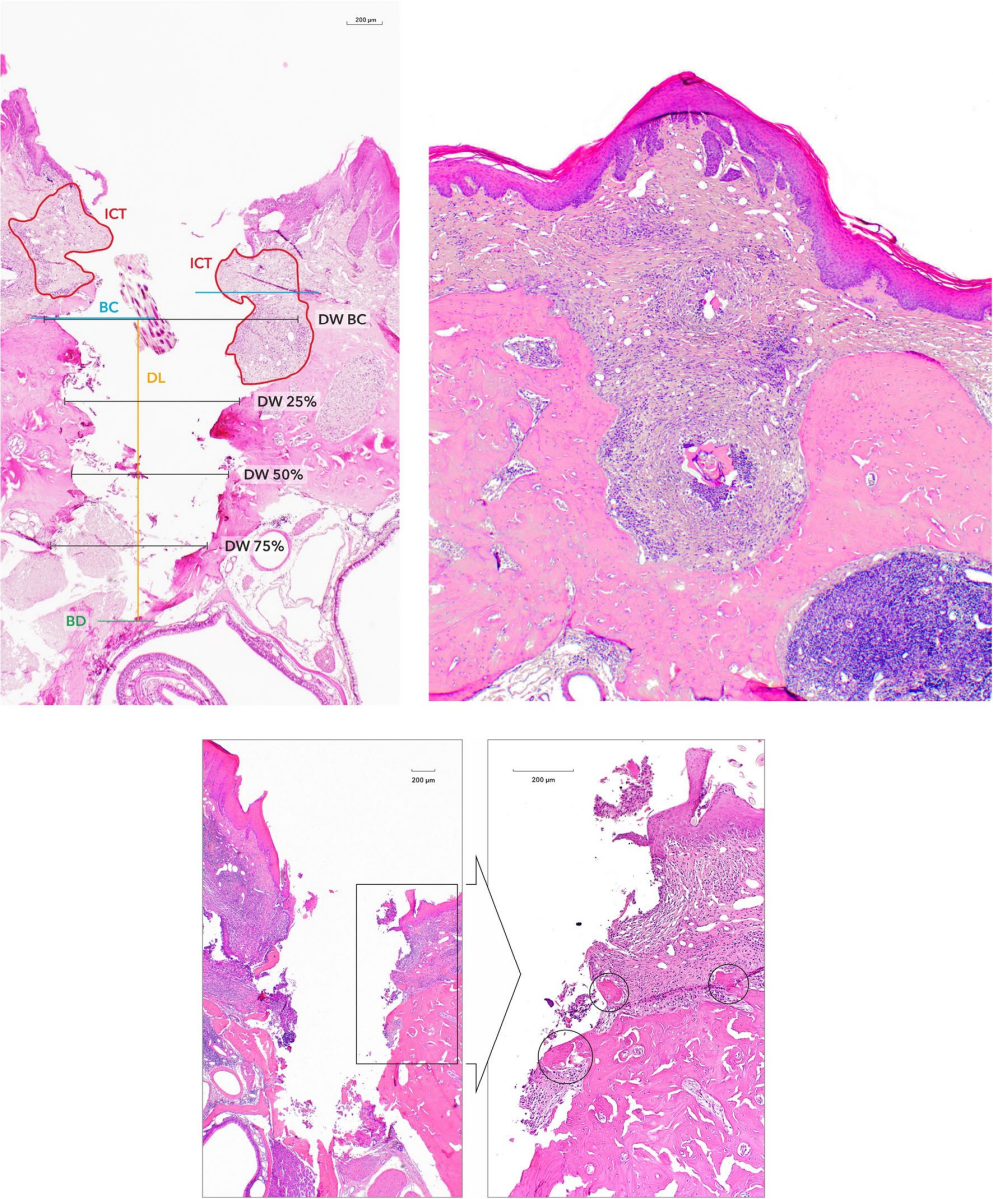
**a** Landmarks and outcomes defined for the histomorphometrical analysis—BC on the vestibular and oral aspects; BD, DL measured from the lower BC to BD; surface area of the ICT, DW measured from the vestibular to oral BC, at the 25%, 50%, and 75% levels of the DL (Zo group). **b** Case presenting the bone sequester (BS) following implant loss during the healing phase after surgical peri-implantitis treatment with OFD approach (De + Be group). **c** Case depicting presence of bone substitute material (Bio-Oss Collagen) following RT peri-implantitis treatment (Zo group)

Serial digital images (BX53, Olympus, Hamburg, Germany) were obtained from each specimen and evaluated using a specialized software (cellSens, Olympus). The following landmarks were identified in the histological sections at each experimental site (Fig. 2a) [21]: the bone crest at the oral and vestibular aspects (BC) and the defect bottom (BD).

The following measurements were assessed:

- Defect length (DL): Measured in millimeters by drawing a vertical line following the long axis of the implant bed from the horizontal line from the lower BC to the BD
- Defect width (DW): Measured in millimeters from the vestibular to oral BC, at the level of 25% of the DL, 50% of the DL, and 75% of the DL
- The surface area (mm^2^) of the inflammatory cell infiltrate (ICT) and, when present, the surface area (mm^2^) of the bone sequester (BS) were assessed using an implemented edge detection tool (Fig. 2b).

One previously calibrated examiner (N.K.) performed all measurements. Calibration was accepted when the intra-examiner correlation coefficient in assessing the repeated measurements of *n* = 5 sections was ≥ 95%.

### Sample size calculation

This analysis is a pilot study, and the sample size was not determined. The present findings will be used for future studies.

### Statistical analysis

The statistical analysis was performed using a commercially available software (SPSS, 19.0, Chicago, IL, USA, R and Rstudio). For each animal, the mean value of the histomorphometrical measurements assessed for two implants was estimated. The mean values, standard deviations, and confidence intervals for each variable (95% CI) were calculated while considering animals as a statistical unit. Considering that both treatment approaches (RT and OFD) were associated with similar results, linear regression analyses assessed only the experimental group (i.e., Co, De, De + Be, Zo, Be, and Zo + Be) for the investigated outcome measures (i.e., DL, DW-BC, 25% DW, 50% DW, 75% DW, ICT, and BS) as a potential predictor for both treatment approaches (RT + OFD). A Shapiro–Wilk test with a significance level of 5% was conducted to assess the normality of the data. When data were not normally distributed, logarithmic transformation was employed. The differences among the experimental groups with respect to implant loss (1) during the progression phase of peri-implantitis and (2) after the treatment were analyzed using logistic regressions. The results were found to be significant (*p* < 0.05).

## Results

Table 1 summarizes the mean DL, DW, ICT, and BS values in different groups. The mean DL in the Co group amounted to 1.07 mm. In the five test groups, the corresponding mean DL values ranged between 0.56 and 1.19 mm, with the lowest values assessed in the Be group and the highest measured in the De + Be group, respectively. The mean BC-DW measurements in the Co group were 1.01 mm, and the corresponding measurements in the test groups in descending order were 1.44 mm (Be + Zo), 1.0 mm (Zo), 0.96 mm (Be), 0.92 mm (De + Be), and 0.86 mm (De).

**Table 1:** Histomorphometrical analysis (mean ± SD; 95% CI) of defect length (DL, mm), defect width (DW, mm), inflamamtory cell infiltarte (ICT, mm^2^), and bone sequester (BS, mm^2^) in different groups are reported on anlimal level (*n* = 21 animals)

Group and treatment approach/number of animals per group	DL (mm)	BC-DW (mm)	25% DW (mm)	50% DW (mm)	75% DW (mm)	ICT (mm^2^)	Number of animals presenting BS/surface area BS (mm^2^)
De RT + OFD (*n* = 5)							
Mean	0.90	0.86	0.72	0.61	0.47	0.71	0.10
SD	0.22	0.18	0.15	0.23	0.25	0.51	0.09
95% -CI	(0.71, 1.1)	(0.7, 1.02)	(0.58, 0.85)	(0.42, 0.81)	(0.25, 0.69)	(0.26, 1.16)	(0.02, 0.18)
De RT (*n* = 3)							
Mean	0.93	0.89	0.90	0.70	0.46	0.81	3/0.053
SD	0.22	0.19	0.19	0.14	0.10	0.70	0.03
95% CI	(0.68, 1.18)	(0.68, 1.11)	(0.64, 0.86)	(0.53, 0.86)	(0.36, 0.58)	(0.02, 1.60)	(0.02, 0.08)
De OFD (*n* = 2)							
Mean	0.87	0.81	0.81	0.49	0.48	0.56	
SD	0.30	0.21	0.21	0.34	0.48	0.09	1/0.23
95% CI	(0.46, 1.28)	(0.52, 1.10)	(0.31, 1.04)	(0.02, 0.96)	(−0.18, 1.14)	(0.42, 0.69)	
De + Be RT + OFD (*n* = 2)							
Mean	1.19	0.92	0.80	0.79	0.69	0.62	0.27
SD	0.31	0.42	0.20	0.23	1.13	0.18	0.15
95% -CI	(0.75, 1.62)	(0.34, 1.5)	(0.53, 1.07)	(0.77, 0.8)	(0.51, 0.87)	(0.37, 0.87)	(0.06, 0.48)
De + Be RT (*n* = 1)	0.97	1.21	0.94	0.80	0.59	0.50	0.16
De + Be OFD (*n* = 1)	1.41	0.63	0.66	0.78	0.78	0.74	0.37
Zo RT + OFD (*n* = 4)							
Mean	0.88	1.00	0.87	0.77	0.69	0.57	0.15
SD	0.18	0.09	0.06	0.08	1.33	0.35	
95% -CI	(0.71, 1.05)	(0.91, 1.09)	(0.82, 0.93)	(0.69, 0.84)	(0.56, 0.82)	(0.22, 0.91)	
Zo RT (*n* = 3)							
Mean	0.94	0.96	0.96	0.75	0.72	0.41	–
SD	0.15	0.058	0.06	0.07	0.15	0.20	
95% CI	(0.76, 1.11)	(0.90, 1.00)	(0.79, 0.95)	(0.66, 0.83)	(0.55, 0.88)	(0.18, 0.64)	
Zo OFD (*n* = 1)	0.69	0.88	0.84	0.60	1.03	0.15	0.69
Be RT (*n* = 1)	0.56	0.96	0.98	0.71	0.48	0.56	–
Zo + Be RT + OFD (*n* = 4)							
Mean	0.90	1.44	1.37	1.29	1.00	1.65	0.23
SD	0.41	0.58	0.59	0.68	0.51	1.11	0.24
95% -CI	(0.5, 1.3)	(0.87, 2)	(0.79, 1.95)	(0.62, 1.96)	(0.5, 1.5)	(0.57, 2.74)	(0, 0.46)
Zo + Be RT (*n* = 2)							
Mean	0.81	1.12	1.12	1.03	0.83	1.26	2/0.09
SD	0.31	0.42	0.42	0.19	0.09	0.29	0.09
95% CI	(0.38, 1.24)	(0.54, 1.69)	(0.79, 1.46)	(0.77, 1.30)	(0.71, 0.96)	(0.86, 1.66)	(−0.03, 0.22)
Zo + Be OFD (*n* = 2)							
Mean	1.0	1.17	1.76	1.55	1.18	2.05	2/0.36
SD	0.61	0.59	0.64	1.05	0.81	1.73	0.30
95% CI	(0.15, 1.83)	(0.87, 2.65)	(0.42, 2.82)	(0.10, 3.00)	(0.05, 2.29)	(−0.34, 4.44)	(−0.05, 0.77)
Control RT + OFD (*n* = 5)							
Mean	1.07	1.01	0.84	0.72	0.53	0.74	0.27
SD	0.34	0.48	0.45	0.34	0.21	0.36	0.36
95%-CI	(0.78, 1.37)	(0.6, 1.43)	(0.45, 1.24)	(0.42, 1.02)	(0.35, 0.71)	(0.42, 1.06)	(−0.04 , 0.59 )
Control RT (*n* = 3)							
Mean	1.09	1.17	1.17	0.78	0.62	0.82	3/0.30
SD	0.39	0.59	0.59	0.23	0.14	0.49	0.43
95% CI	(0.64, 1.53)	(0.51, 1.84)	(0.39, 1.52)	(0.52, 1.04)	(0.46, 0.78)	(0.27, 1.37)	(−0.19, 0.79)
Control OFD (*n* = 2)							
Mean	1.05	0.77	0.77	0.64	0.40	0.62	1/0.18
SD	0.38	0.15	0.15	0.59	0.28	0.12	
95% CI	(0.53, 1.56)	(0.57, 0.97)	(0.03, 1.32)	(−0.18, 1.45)	(0.01, 0.79)	(0.46, 0.79)	

*RT* reconstructive peri-implantitis treatment, *OFD* open flap debridement, *Zo* zoledronate, *De* denosumab, *Be* bevacizumab, *CI* confidence interval, *SD* standard deviation

In all groups, there was a tendency toward a gradual reduction of DW values from 25% DW to 75% DW reference points. The mean 25% DW value in the Co group was 0.84 mm. In descending order, the respective measurements in the test groups amounted to 1.37 mm (Be + Zo), 0.98 mm (Be), 0.87 mm (Zo), 0.8 mm (De + Be), and 0.72 mm (De). The mean 50% DW value in the Co groups amounted to 0.72 mm and ranged between 1.29 and 0.61 mm in the test groups, with the highest measurement assessed in the Be + Zo group and the lowest in the De group, respectively. The mean 75% DW value in the Co group was 0.53 mm. The respective measurements in the test group varied between 1.0 and 0.47 mm, with the highest measurement found in the Be + Zo group and the lowest in the De group.

All groups revealed the presence of a chronic-type inflammatory cell infiltrate (ICT). The mean ICT values in the Co group amounted to 0.74 mm^2^. In the test groups, the corresponding values in descending order were 1.65 mm^2^ (Zo + Be), 0.71 mm^2^ (De), 0.62 mm^2^ (De + Be), 0.57 mm^2^ (Zo), and 0.56 mm^2^ (Be).

In total, 4 out of the 5 animals in the Co groups and 11 out of the 16 animals in the test groups revealed isolated BS. No BS could be observed in the Zo (3 animals) and Be (1 animal) groups treated with the RT approach. In the Co group, the BS area amounted to 0.27 mm^2^. In the test groups, the BS ranged between 0.10 mm^2^ (De) and 0.69 mm^2^ (Zo).

The linear regression analysis revealed 1.73 times greater (73% higher) 25% DW measurements in the Zo + Be group compared with the Co group (*p* = 0.04). Likewise, when compared with the Co group, the Zo + Be group showed significantly higher 75% DW values by 0.47 mm (*p* = 0.03). No other significant associations could be observed in the assessed outcome measures with respect to the experimental group.

During the peri-implantitis progression phase, the highest implant loss was registered in the Be group followed by the De + Be group. After peri-implantitis treatment, higher implant loss was documented in the De and Zo + Be groups. The logistic regression did not show significant differences among the groups with respect to implant loss during the progression phase (*p* = 0.07) or after the treatment (*p* = 0.35).

## Discussion

The aim of the present analysis was to investigate the influence of various antiresorptive and antiangiogenic medications on the resolution of experimentally induced peri-implantitis lesions treated with or without adjunctive reconstructive measures (i.e., OFD or RT). The histomorphometric analysis revealed significantly higher 25% DW and 75% DW measurements in the Zo + Be group compared to the Co group, whereas no significant differences were found for the remaining outcome measures among the experimental groups. The latter finding basically aligns with the outcome of one recent experimental study that investigated the influence of antiresorptive/antiangiogenic medications on the extension of experimentally induced peri-implantitis lesions in a rodent model and encompassed analogous experimental groups [21]. In fact, after 16 weeks of peri-implantitis induction, the findings depicted a tendency toward the highest DW values in the BC region in the Zo + Be test group [21]. Given the aforementioned findings, one may assume that the combination of bisphosphonates and antiangiogenic medications (i.e., Zo + Be) associates with larger peri-implant bone defects that consequently appear more difficult to resolve. The adjunctive reconstructive measures were not found to have a beneficial effect on the direct resolution, as no differences were observed between the RT and OFD treatment approaches.

Upon further analysis of the present data set, all implant sites showed the presence of ICT, potentially suggesting that complete disease resolution could not be achieved with either treatment modality (i.e., RT or OFD). This finding corroborates the former preclinical analyses in a canine model in which surgically treated experimental peri-implantitis lesions with various implant surface cleaning methods showed the presence of residual ICT [26, 27]. In line with the present findings, similar ICT values were detected in the peri-implant tissues irrespective of the treatment (i.e., RT or OFD) [27]. Nonetheless, confirmation of the inflammatory nature of the ICT requires detailed immunological analyses, which were not part of the present study. Furthermore, the histological outcomes were not correlated with clinical signs of inflammation (i.e., the presence of bleeding, suppuration) due to limitations in the validity of clinical outcome measurements in a rodent model.

When interpreting the present findings, it should be noted that the largest ICT surface area was observed in the Zo + Be group, which in turn showed the largest residual bone defects depicted by the highest 25% and 75% DW measurements. Although Zo primarily targets osteoclasts by suppressing their resorptive function, it is also known to activate inflammatory signals and induce proinflammatory effects [28–30]. More specifically, Zo has been shown to upregulate the function of M1 macrophages, subsequently resulting in the production of proinflammatory cytokines [28, 29]. The administration of Be was previously linked with a significantly increased risk of infectious events, namely, severe febrile neutropenia, and fistulae/abscesses [31]. Accounting for the infectious events related to the administration of Be and Zo, the highest ICT values measured in the Zo + Be (i.e., bisphosphonate + antiangiogenic medications) group might be at least partially associated with the effect of the medications

Interestingly, except for the Be and Zo groups treated with RT, the presence of BS could be detected in the remaining groups investigated. The isolated sequestered bone fragments were previously reported following the induction of the peri-implantitis lesions [21]. On the other hand, contrary to the former findings, osteonecrosis zones could only seldom be observed, whereas the experimental model in mice reported frequent osteonecrosis detected following either the experimental induction of periodontitis [32] or tooth extraction with or without peri-apical pathologies under the administration of either Zo or RANKL inhibitors [33, 34].

The present study used a “ligature-induced” defect model that has been recently established as a standard experimental model to investigate the pathogenesis and the therapy of peri-implantitis [35]. In animal studies, submarginal placement of ligatures around implants resulted in changes in the composition of the local microbiota and resulted in a subsequent inflammatory destruction of peri-implant tissues [36–38]. The adjunctive application of the LPS booster was shown to considerably contribute to the generation of a more complex immune response, including the occurrence of an earlier and denser local inflammatory infiltrate for the periodontitis induction in rats, in comparison with the use of ligature alone [39]. In fact, the submarginal ligature placement alone failed to induce any significant inflammation and alveolar bone loss in germ-free rats and mice, thus depicting that the inflammation and bone loss sustained during the ligature model are dependent on an adjunctive bacterial challenge [40].

It needs to be noted that, regrettably, more than half of the animals were lost during the progression phase of peri-implantitis. Although the loss of animals could not be related with the experimental procedure, considering the physiological aging of rats and concomitant underlying aging-related pathologies, it could be possible that to some extent the experimentally induced peri-implantitis lesions contributed to the cause of death [41]. Nonetheless, the loss of animals was equally distributed among the test and control groups, which does not support the influence of antiresorptive/antiangiogenic therapy’s administration on the cause of death. In fact, the rate of animal loss in this study is comparable to that reported in one former experimental analysis in rats that employed an analogous model for the induction of peri-implantitis lesions [21].

Although statistically significant differences were absent, there was a tendency toward higher implant loss during the peri-implantitis progression phase in the Be and De +Be groups and in the De and Zo + Be groups after peri-implantitis therapy. Consequently, the limited number of animals available for the analysis might have been the reason for the failure to detect differences between the RT and OFD therapeutic approaches. One might thus assume that a trend for higher implant loss noted in test groups, at least to some extent, is attributable to the administration of the medications. Another factor that might have contributed to the high rate of implant loss was the use of very short smooth-surfaced implants. The use of rough-surfaced implants might have reduced the rates of implant loss, which on the other hand might have aggravated implant removal prior to histological processing. Future preclinical and clinical studies employing various implant micro- and macro-designs are needed for a better understanding of peri-implantitis treatment outcomes under the administration of antiresorptive/antiangiogenic medications.

In conclusion, the present analysis failed to observe any remarkable effects of various antiresorptive/antiangiogenic medications on the resolution of peri-implantitis-induced peri-implantitis lesions, regardless of the surgical approach employed (OFD and RT).
